# On the Nature of the Corpora Lutea, and on the Difference Which They Present According as the Expulsion of the Ovule Has Been Followed or Not by Conception

**Published:** 1845-06

**Authors:** 


*On the Nature of the Corpora Lutea, and on the differences which they present according as the expulsion of the ovule has been followed or not by conception.* By M. Raciborski.—M. Raciborski, from his experiments and dissections relative to the formation of the *corpora lutea*, draws the following conclusions :—

1. The *corpora lutea* are the result of a true hypertrophy of the granular layer which covers the internal membrane of the Graafian vesicles. The anatomical elements of these two parts are absolutely the same, only the granulations of the *corpora lutea* are much more numerous, and involve many more oily globules of a yellow colour.

2. The transformation of the internal tunic into *corpus luteum* commences before the rupture of the vesicle, at the moment when it is ready to give passage to the ovule.

3. As soon as the Graafian vesicles are ruptured the transformation of the internal membrane into *corpus luteum* acquires an extraordinary activity. But there are here two essential differences remarked according as the expulsion of the ovule has been spontaneous, as occurs at each menstrual and rutting period, or according as it has been attended with sexual intercourse and conception.

In the females of most of our domestic animals, as the pig, cow, sheep, &c., this difference does not exist. Whether these animals have or have not had connection with the males, the expulsion of the ovule is always followed by the formation of *corpora lutea*, represented by fleshy masses of a yellow or reddish colour. It is different, however, with women. If the expulsion of the ovule is not followed by conception, as happens at the ordinary menstrual periods, then these granulations increase in size and number ; but this activity of nutrition soon stops and proceeds no farther than the formation of a thin membrane of a more or less deep yellow colour applied against the proper membrane of the vesicles ; this membrane surrounds a cavity in which may generally be found traces of a clot of blood. If, on the other hand, conception coincides with the expulsion of the ovule, the elements of the granular tunic augment so in number and volume, that in a short time they form a pretty voluminous mass, which of itself fills the whole cavity of the vesicle.

4. In all women delivered at the full time, we find a *corpus luteum*, such as we have described. But what is very remarkable is the rapidity with which the *corpus luteum* decreases and becomes atrophied as soon as delivery takes place. Thus a *corpus luteum* which on the second or third day after delivery, would have a medium diameter of 7-10ths of an inch, by the tenth day would be reduced more than one-half ; and, by the end of three months, a small scarcely coloured particle, not exceeding a line in diameter, could alone be detected.

5. It results from this, that in women it is very easy to distinguish, by the inspection alone, cases of the spontaneous expulsion of ova from those in which the expulsion has been followed by conception.—*Edinburgh Med. and Surg. Journ., from Bulletin de l'Academie Royale de Medicine.*

